# Butterbur Leaves Attenuate Memory Impairment and Neuronal Cell Damage in Amyloid Beta-Induced Alzheimer’s Disease Models

**DOI:** 10.3390/ijms19061644

**Published:** 2018-06-01

**Authors:** Namkwon Kim, Jin Gyu Choi, Sangsu Park, Jong Kil Lee, Myung Sook Oh

**Affiliations:** 1 Department of Life and Nanopharmaceutical Sciences, Graduate School, Kyung Hee University, 26, Kyungheedae-ro, Dongdaemun-gu, Seoul 02447, Korea; kop03@khu.ac.kr; 2 BK21 PLUS Integrated Education and Research Center for Nature-Inspired Drug Development Targeting Healthy Aging, Kyung Hee University, 26, Kyungheedae-ro, Dongdaemun-gu, Seoul 02447, Korea; choijg2002@khu.ac.kr; 3 NATUREBIO Co., Ltd., 132, Pureunsol Culture Hall, 26, Kyungheedae-ro, Dongdaemun-gu, Seoul 02447, Korea; x-zara@nate.com; 4 Department of Pharmacy, College of Pharmacy, Kyung Hee University, 26 Kyungheedae-ro, Dongdaemun-gu, Seoul 02447, Korea; 5 Department of Oriental Pharmaceutical Science, College of Pharmacy, Kyung Hee University, 26, Kyungheedae-ro, Dongdaemun-gu, Seoul 02447, Korea

**Keywords:** Alzheimer’s disease, butterbur, *Petasites japonicus*, amyloid beta, memory

## Abstract

Alzheimer’s disease (AD) is the most prevalent neurodegenerative disease, and is characterized by the accumulation of amyloid beta (Aβ) as a pathological hallmark. Aβ plays a central role in neuronal degeneration and synaptic dysfunction through the generation of excessive oxidative stress. In the present study, we explored whether leaves of *Petasites japonicus* (Siebold & Zucc.) Maxim. (PL), called butterbur and traditionally used in folk medicine, show neuroprotective action against Aβ_25–35_ plaque neurotoxicity in vitro and in vivo. We found that PL protected Aβ_25–35_ plaque-induced neuronal cell death and intracellular reactive oxygen species generation in HT22 cells by elevating expression levels of phosphorylated cyclic AMP response element-binding protein, heme oxygenase-1, and NAD(P)H quinine dehydrogenase 1. These neuroprotective effects of PL were also observed in Aβ_25–35_ plaque-injected AD mouse models. Moreover, administration of PL diminished Aβ_25–35_ plaque-induced synaptic dysfunction and memory impairment in mice. These findings lead us to suggest that PL can protect neurons against Aβ_25–35_ plaque-induced neurotoxicity and thus may be a potential candidate to regulate the progression of AD.

## 1. Introduction

Alzheimer’s disease (AD), an irreversible and degenerative brain disease, is characterized by progressive memory and cognitive dysfunction [1,2]. The hallmarks of AD are senile plaque by excessive accumulation of extracellular amyloid beta (Aβ) and neurofibrillary tangles by hyperphosphorylation of intracellular tau in the brain [3]. Although the exact pathology of AD is not yet clear, a number of studies have shown that Aβ in the brain is the primary cause of the pathogenesis of AD. A correlation between Aβ accumulation and memory impairment in AD patients has also been reported [4,5,6]. Aβ induces the generation of proapoptotic factors such as cytochrome c and caspase-3 by increasing intracellular calcium levels and facilitating the formation of reactive oxygen species (ROS) [7]. Accumulating evidence suggests that increased ROS by Aβ is one of the most important mechanisms involved in the neuronal degeneration, synaptic loss, and memory impairment of AD [8]. Thus, inhibition of Aβ-induced neurotoxicity may provide neuroprotective benefits in AD.

Butterbur, *Petasites japonicus* (Siebold & Zucc.) Maxim., has been known for various biological activities, including antiallergic, antiinflammatory, and antioxidant effects [9,10,11]. *P. japonicus* leaf (PL) extract and petaslignolide A, an active compound of PL, have been reported to have neuroprotective effects on oxidative stress induced by kainic acid in mice [12,13]. In addition, kaempferol isolated from *P. japonicus* stems reduced the damage of neuronal cells against glutamate-induced oxidative stress by regulating antiapoptotic factors (B-cell lymphoma 2), proapoptotic factors (Bax-like BH3 protein and apoptosis-inducing factor), and mitogen-activated protein kinases (p38, extracellular signal-regulated kinase, and c-Jun N-terminal kinase) in mouse hippocampal cells [14]. Park et al. reported that both whole extract of *P. japonicus* and two isolated compounds, petatewalide B and bakkenolide B, inhibited neuroinflammatory responses in lipopolysaccharide-stimulated BV2 microglia cells via upregulation of AMP-activated protein kinase/nuclear factor erythroid 2-related factor 2 signaling [15,16]. Also, flower buds of *P. japonicus* inhibited aggregation of bovine serum albumin and lactalbumin as well as Aβ_1–42_ [17]. However, the neuroprotective effects of PL in Aβ-induced AD in in vitro and in vivo models have not been explored.

Based on these concepts and findings, the present study was designed to determine whether PL has neuroprotective effects against Aβ_25–35_ plaque-induced neurotoxicity, and if so, how it might lead to beneficial effects in AD. To explore this, we used HT22 cells treated with Aβ_25–35_ plaque and acutely induced AD mouse models.

## 2. Results

### 2.1. Standardization of PL

**Figure 1:**
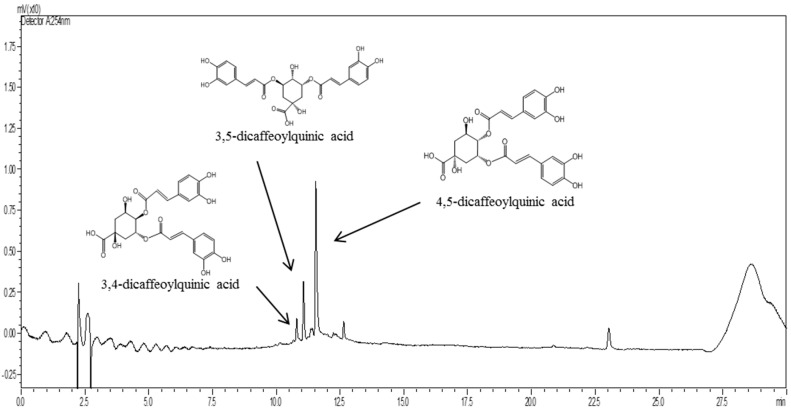
Representative HPLC-MS chromatogram of 3,4-dicaffeoylquninic acid, 3,5-dicaffeoylquinic acid, and 4,5-dicaffeoylquninic acid in *Petasites japonicus* leaf (PL).

To determine the active constituents of PL, it was examined by high performance liquid chromatography-mass spectrometry (HPLC-MS) analysis. We observed that PL contains 3,4-dicaffeoylquinic acid (0.02 μg/g), 3,5-dicaffeoylquinic acid (0.15 μg/g), and 4,5-dicaffeoylquinic acid (0.43 μg/g; Figure 1). These compounds were used to standardize the PL in this study. These compounds were shown to have antioxidant effects in previous studies [18,19,20].

### 2.2. PL Protects Neuronal Cells against Aβ_25–35_ Plaque Toxicity

**Figure 2:**
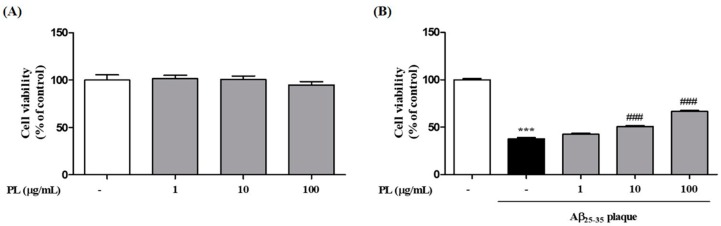
Effect of PL on cell death induced by Aβ_25–35_ plaque in HT22 cells. HT22 cells were treated with PL (1–100 μg/mL) for 1 h and incubated (**A**) without or (**B**) with Aβ_25–35_ plaque (10 μmol/L) for a further 23 h. Cell viabilities were measured by 3-(4,5-dimethylthiazol-2-yl)-2,5-diphenyltetrazolium bromide (MTT) assay 24 h after sample treatment and are presented as percentage of control. Values are mean ± standard error of the mean. *** *p* < 0.001 vs. control group, ### *p* < 0.001 vs. Aβ_25–35_ plaque group.

We first checked the cytotoxicity of PL in HT22 cells. Treatment with PL at 1, 10, and 100 μg/mL for 24 h did not show toxicity to HT22 cells (Figure 2A). To investigate whether PL protects neuronal cells from Aβ_25–35_ plaque, HT22 cells were treated with Aβ_25–35_ plaque in the presence or absence of PL for 24 h. After exposure to Aβ_25–35_ plaque, cell viability was significantly decreased to 37.69 ± 1.21% compared with the control group, whereas PL at 10 and 100 μg/mL significantly restored cell viability to 50.60 ± 1.08% and 66.61 ± 1.00%, respectively (Figure 2B).

### 2.3. PL Reduces the Accumulation of ROS Induced by Aβ_25–35_ Plaque via Antioxidant Responses

**Figure 3:**
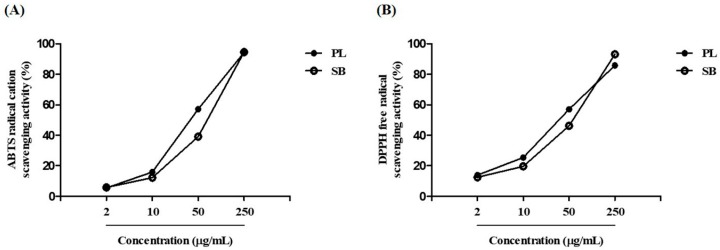
Radical scavenging activity of PL. (**A**) 2,2-azinobis-(3-ethyl-benzthiazolin-6-sulphonic acid) (ABTS) radical cation and (**B**) 2,2-diphenyl-2-picrylhydrazyl (DPPH) free radical scavenging activities at concentrations of 2–250 μg/mL. SB, *Scutellaria baicalensis*.

Oxidative stress occurs when ROS levels exceed the antioxidant capacity of cells [21]. To evaluate the scavenging activity of PL on free radicals, which induce oxidative damage to biomolecules, we performed 2,2-azinobis-(3-ethyl-benzthiazolin-6-sulphonic acid) (ABTS) and 2,2-diphenyl-2-picrylhydrazyl (DPPH) assays. In these two assays, PL showed dose-dependent free radical scavenging activity (Figure 3). The inhibiting concentration (IC_50_) values of ABTS and DPPH for PL were 38.84 and 35.89 μg/mL, respectively. These data suggest that PL exhibits potential scavenging activities against free radicals compared with *Scutellaria baicalensis* extract, used as a positive control.

**Figure 4:**
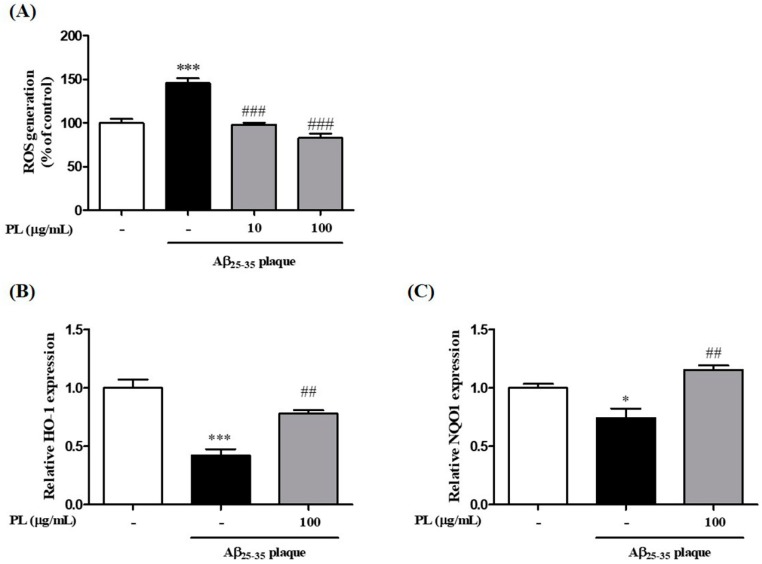

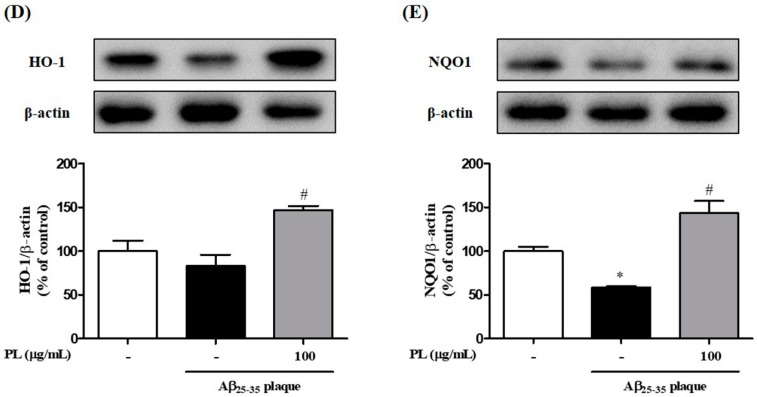
Inhibitory effect of PL on intracellular reactive oxygen species (ROS) generation induced by Aβ_25–35_ plaque in HT22 cells. Cells were treated with PL (10 and 100 μg/mL) for 1 h and incubated with Aβ (10 μmol/L) for a further 23 h. (**A**) ROS generation was measured by fluorescence intensity of 2’,7’-dichlorofluorescin (DCF) 24 h after PL treatment and is presented as percentage of control. Cell lysates were assessed by qRT-PCR and Western blot using heme oxygenase-1 (HO-1) and NAD(P)H quinine dehydrogenase 1 (NQO1) antibodies. (**B**–**E**) Quantification of (**B**) mRNA and (**D**) protein of HO-1 and (**C**) mRNA and (**E**) protein of NQO1. The data were normalized to GAPDH or β-actin. Values are mean ± standard error of the mean. * *p* < 0.05 and *** *p* < 0.001 vs. control group; # *p* < 0.05, ## *p* < 0.01 and ### *p* < 0.001 vs. Aβ_25–35_ plaque group.

We then investigated whether PL inhibits the increase of Aβ_25–35_ plaque-induced intracellular ROS levels in HT22 cells. Aβ_25–35_ plaque significantly increased ROS levels by 145.84 ± 5.23% compared with the control group. As shown in Figure 4A, this increase was reduced by pretreatment with PL in HT22 cells in a concentration-dependent manner (10 μg/mL: 98.01 ± 2.27%; 100 μg/mL: 83.00 ± 4.81%). To explore the underlying mechanism by which PL reduced ROS levels in Aβ_25–35_ plaque-stimulated HT22 cells, we measured protein and mRNA expression levels of heme oxygenase-1 (HO-1) and NAD(P)H quinine dehydrogenase 1 (NQO1), which have pivotal roles in cellular antioxidant signaling [22,23]. The mRNA expression levels of HO-1 and NQO1 were decreased by Aβ_25–35_ plaque, but pretreatment with PL significantly ameliorated that effect (Figure 4B,C). In a parallel experiment, we analyzed the protein expression levels of HO-1 and NQO1. As with mRNA expression, PL significantly increased the protein expression levels of HO-1 and NQO1 reduced by Aβ_25–35_ plaque (Figure 4D,E).

### 2.4. PL Increases Phosphorylation of CREB in Aβ_25–35_ Plaque-Stimulated HT22 Cells and Mice

**Figure 5:**
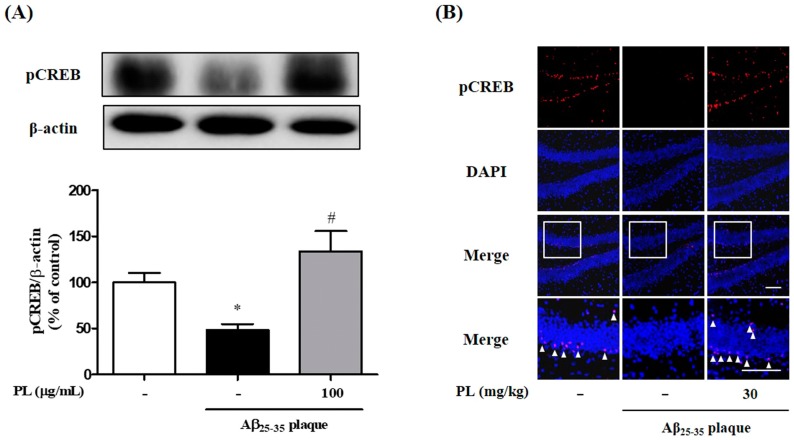
Effect of PL on phosphorylation of cyclic AMP response element-binding protein (CREB) in Aβ_25–35_ plaque-stimulated HT22 cells and mice. Cells were treated with PL (100 μg/mL) for 1 h and incubated with Aβ (10 μmol/L) for a further 23 h. Cell lysates were assessed by Western blot using pCREB antibody. (**A**) Quantification of pCREB protein was normalized to β-actin. After stereotaxic injection of Aβ_25–35_ plaque, mice were treated with vehicle or PL for 17 days; (**B**) pCREB expression was explored in the dentate gyrus (DG). Scale bar = 100 μm. Values are mean ± standard error of the mean. * *p* < 0.05 vs. control group; # *p* < 0.05 vs. Aβ_25–35_ plaque group. The arrowheads in magnified images point to pCREB/4’,6-diamidino-2-phenylindole (DAPI) double-labeled neurons.

ROS induced by Aβ regulates the expression of cyclic AMP response element-binding protein (CREB), which is closely related to neuronal survival, synaptic plasticity, memory function, and antioxidant systems [24,25,26]. It has been reported that antioxidant defense systems attenuated ROS-mediated damage of neuronal cells [26]. We evaluated whether PL treatment activates CREB signaling. Aβ_25–35_ plaque decreased phosphorylation of CREB in HT22 cells compared with the control group. PL at 100 μg/mL significantly reversed this phenomenon (Figure 5A). Next, to confirm the in vitro results, we also investigated expression of pCREB in the dentate gyrus (DG) of Aβ_25–35_ plaque-injected mice using immunofluorescence analysis. Aβ_25–35_ plaque-induced decline of pCREB expression was prevented by PL administration (Figure 5B). These findings suggest that PL decreased Aβ_25–35_ plaque-induced intracellular ROS accumulation via upregulation of pCREB and antioxidant enzymes.

### 2.5. PL Protects Hippocampal Cells against Aβ_25–35_ Plaque Toxicity in Mice

**Figure 6:**
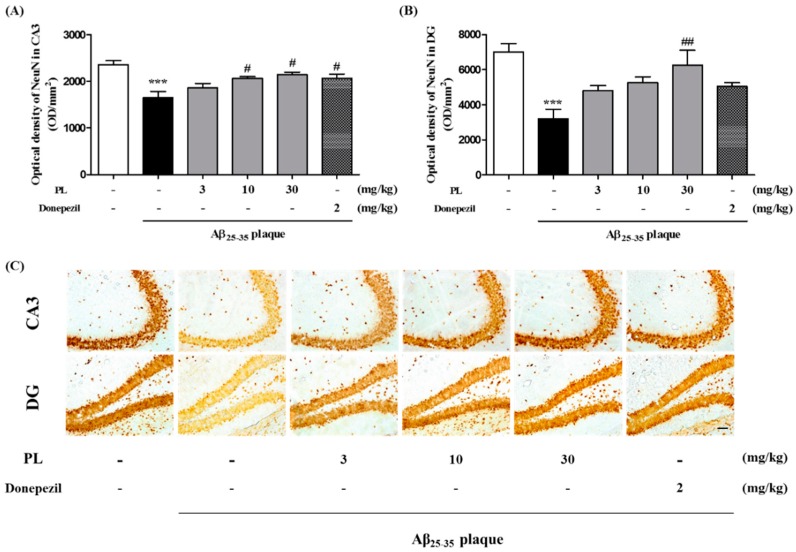
Effect of PL on neuronal cell loss in Aβ_25–35_ plaque-injected mice. After stereotaxic injection of Aβ_25–35_ plaque, mice were treated with vehicle, PL, or donepezil for 17 days. Neuronal cells were stained with anti-NeuN antibody. The optical density of the NeuN-stained cells was measured in the (**A**) CA3 and (**B**) DG; (**C**) Representative images of NeuN-positive neurons in the CA3 (up) and DG (down) of mice brain. Scale bar = 30 μm. Values are mean ± standard error of the mean. *** *p* < 0.001 vs. sham group; # *p* < 0.05 and ## *p* < 0.01 vs. Aβ_25–35_ plaque group. Donepezil was used as a positive control.

Our in vitro data indicate that PL can protect neuronal cells against the oxidative stress induced by Aβ_25–35_ plaque. To confirm whether PL has neuroprotective effects in the AD mouse model, we performed neuronal nuclei (NeuN) immunohistochemistry in the mouse hippocampus. NeuN is a nuclear protein used as a neuronal marker [27]. The optical density of NeuN in the hippocampal CA3 and DG regions of the Aβ_25–35_ plaque group (1648.33 ± 135.81 and 3213.15 ± 531.84 OD/mm^2^, respectively) was significantly lower than that of the sham group. Treatment with PL at 30 mg/kg significantly increased NeuN-positive cells compared with the Aβ_25–35_ plaque group (Figure 6). These data demonstrate that PL protects neuronal cells from Aβ toxicity in the hippocampus.

### 2.6. PL Inhibits Aβ_25–35_ Plaque-Induced Synaptotoxicity in Mice

**Figure 7:**
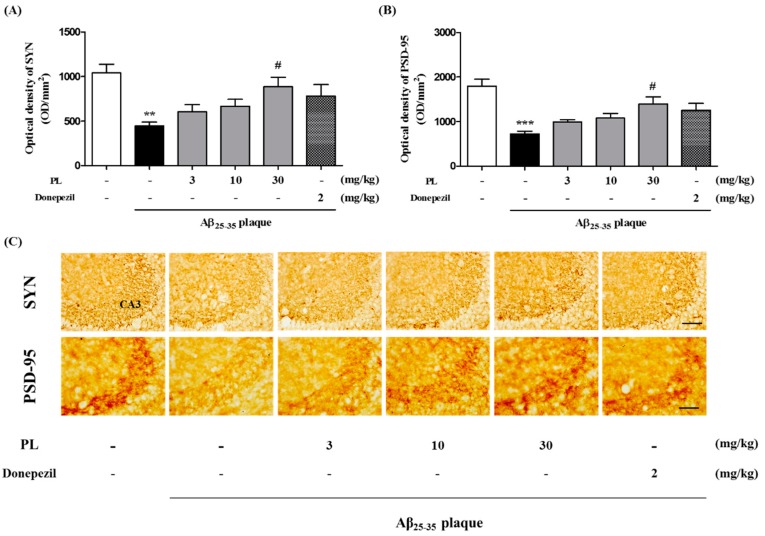
Effect of PL on synaptotoxicity in Aβ_25–35_ plaque-injected mice. After stereotaxic injection of Aβ_25–35_ plaque, mice were treated with vehicle, PL, or donepezil for 17 days. Synapses were stained with anti-SYN antibody or anti-PSD-95 antibody. The optical density of the SYN (**A**) and PSD-95 (**B**) were measured in the CA3; (**C**) Representative images of SYN (up) and PSD-95 (down) in the CA3 of mice brain. Scale bar = 30 μm. Values are mean ± standard error of the mean. ** *p* < 0.01 and *** *p* < 0.001 vs. sham group; # *p* < 0.05 vs. Aβ_25–35_ plaque group. Donepezil was used as a positive control.

It has been shown that accumulated Aβ causes synaptic degeneration involved in memory function [28,29]. We examined the effect of PL on synaptotoxicity induced by Aβ_25–35_ plaque. Synaptophysin (SYN) and postsynaptic density protein 95 (PSD-95) as synaptic markers are closely connected with cognitive function, and altering of these markers is important for synaptic connectivity and plasticity [28]. In our data, the immunoreactivity of both SYN and PSD-95 was significantly decreased in the hippocampal CA3 region of the Aβ_25–35_ plaque group (447.10 ± 42.62 and 726.68 ± 59.99 OD/mm^2^) compared with the sham group. PL at 30 mg/kg (887.86 ± 105.81 and 1397.81 ± 154.33 OD/mm^2^) significantly increased the intensity of SYN and PSD-95 immunoreactivity compared with the Aβ_25–35_ plaque group (Figure 7).

### 2.7. PL Ameliorates Memory Impairment Induced by Aβ_25–35_ Plaque

**Figure 8:**
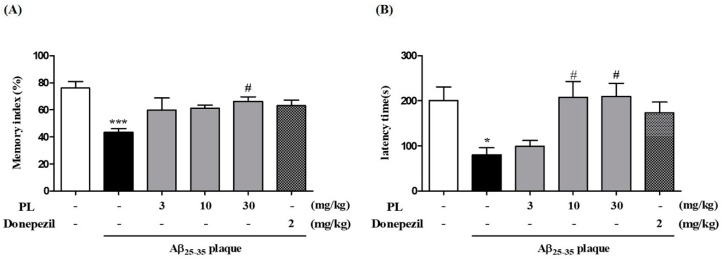
Effect of PL on memory impairment induced by Aβ_25–35_ plaque in mice, as measured by (**A**) novel object recognition test (NORT) and (**B**) passive avoidance test (PAT) (*n* = 7–8 per group). Values are mean ± standard error of the mean. * *p* < 0.01 and *** *p* < 0.001 vs. sham group; # *p* < 0.05 vs. Aβ_25–35_ plaque group. Donepezil was used as a positive control.

To explore the effect of PL on Aβ_25–35_ plaque-induced cognitive deficits, we performed a novel object recognition test (NORT) and a passive avoidance test (PAT). In the NORT, the Aβ_25–35_ plaque group (43.25 ± 2.86%) spent less time exploring the novel object than the sham group (76.12 ± 4.89%). The memory index of PL at 30 mg/kg was significantly increased by 66.32 ± 3.26% compared with the Aβ_25–35_ plaque group (Figure 8A). The Aβ_25–35_ plaque group also exhibited fear memory impairment (80.45 ± 15.31 s) compared with the sham group (200.95 ± 30.11 s) in the PAT. However, the latency time after treatment with PL at 10 and 30 mg/kg was significantly increased in the test trial (207.91 ± 34.99 and 209.89 ± 28.88 s, respectively; Figure 8B). These data suggest that PL might alleviate memory deficits induced by Aβ_25–35_ plaque by inhibiting neurodegeneration and synaptic disruption in the mouse hippocampus.

## 3. Discussion

In this study, we demonstrated that PL has neuroprotective effects in AD models. PL could prevent hippocampal cell death from Aβ_25–35_ plaque by regulating ROS generation, CREB phosphorylation, and antioxidant enzymes. Furthermore, PL alleviated memory impairment by protecting neuronal cells and synapses from Aβ_25–35_ plaque in mice.

Oxidative stress induced by Aβ toxicity plays a key role in the pathogenesis of AD and is involved in intracellular ROS formation and antioxidative system dysfunction [30]. Aβ-induced oxidative stress can also induce the loss of synapses and neuronal cells through inhibition of CREB phosphorylation [31]. It has been reported that phosphorylation of CREB activates HO-1 and NQO1, which protect hippocampal cells against glutamate-induced damage [32]. CREB is a nuclear protein that performs a critical role in cell survival as well as memory formation. CREB can influence memory by inducing neuronal excitability and synaptic modification, because long-term potentiation depends on the depolarization level of postsynaptic neurons [33]. Our results suggest that PL inhibits neuronal cell death by Aβ_25–35_ plaque-induced ROS by modulating CREB phosphorylation-mediated generation of antioxidant enzymes (HO-1 and NQO1). In accordance with our in vitro results, we confirmed the free radical scavenging effects of PL in ABTS and DPPH assays; IC_50_ values of PL-treated groups were lower than those of the positive controls. Although the exact compounds within PL evoking neuroprotective effects remain unclear, some previous studies may partly explain our results. It has been reported that 3,4-dicaffeoylquinic acid, 3,5-dicaffeoylquinic acid, and 4,5-dicaffeoylquinic acid, which are standardized compounds of PL in this study, displayed radical scavenging actions in ABTS assays [18]. In addition, 3,4-dicaffeoylquinic acid and 3,5-dicaffeoylquinic acid protected primary cortical neurons from glutamate-induced apoptosis through regulation of calcium influx and ROS levels [19]. 3,5-Dicaffeoylquinic acid also protected neuronal cells against H_2_O_2_-induced oxidative stress via inhibition of caspase-3 activation and modulation of glutathione levels [20].

Synaptic connections sculpted by synaptic plasticity are strongly associated with memory formation. SYN is a synaptic vesicle protein involved in Ca^2+^-mediated neurotransmitter release [28]. PSD-95 is localized in the postsynaptic density of brain neurons and organizes the composition and structure of synaptic proteins [28]. It has been shown that Aβ accelerates synapse failure by axonal and dendritic injury, leading to memory impairment [34,35]. Interestingly, we found that PL inhibited neuronal cell death and synaptic loss in AD mice. Also, PL resulted in amelioration of memory impairment in these mice. Although exact mechanisms need to be further investigated, our findings suggest that PL can reduce Aβ_25–35_ plaque-mediated AD symptoms by protecting hippocampal neurons and synapses. Similar to our results, 3,5-dicaffeoylquinic acid within PL improved spatial learning and memory in senescence-accelerated prone mice through induction of glycolytic enzyme (phosphoglycerate kinase-1) mRNA expression and ATP production [36]. These compounds may contribute to pharmacological activities of PL on AD.

## 4. Materials and Methods

### 4.1. Materials

Dulbecco’s Modified Eagle Medium (DMEM), fetal bovine serum (FBS), and penicillin-streptomycin (PS) were purchased from Hyclone Laboratories (Logan, UT, USA). Aβ_25–35_ peptide, dimethylsulfoxide anhydrous (DMSO), mouse anti-SYN, triton X-100, slide mounting medium for histology, paraformaldehyde (PFA), 3,3-diaminobenzidine (DAB), DPPH, ABTS, 3-(4,5-dimethylthiazol-2-yl)-2,5-diphenyltetrazolium bromide (MTT), 2,7-dichlorodihydrofluorescein diacetate (DCFH-DA), phosphate buffer (PB), and phosphate buffered saline (PBS, pH 7.4) were purchased from Sigma Aldrich (St. Louis, MO, USA). Rabbit anti-PSD-95 and goat anti-NQO1 were purchased from Abcam (Cambridge, UK). Rabbit anti-HO-1 was purchased from Enzo Life Sciences (Farmingdale, NY, USA). Rabbit anti-phosphorylated CREB (pCREB) was purchased from Santa Cruz Biotechnology (Santa Cruz, CA, USA). Mouse anti-NeuN was purchased from Millipore (Billerica, MA, USA). Normal goat serum (NGS), avidin-viotin complex (ABC), and biotinylated anti-mouse and anti-rabbit antibodies were purchased from Vector Lab (Burlingame, CA, USA).

### 4.2. Preparation and Standardization of PL

Fresh PL was purchased from a local market (Yeosu, Korea) and dried in an air dryer at 50 °C for 24 h. The leaves were then boiled with a 20-fold quantity of distilled water at 90 °C for 4 h. The obtained extract was filtered, concentrated, and freeze-dried to yield powder (yield: 25.0%). Next, the powder was mixed with a twofold quantity of 70% ethanol at room temperature for 24 h. Its sediment was freeze-dried to yield powder (yield: 12.7%). The powder was stored at −20 °C for further use.

3,4-Dicaffeoylquninic acid (5 mg), 3,5-dicaffeoylquinic acid (46 mg), and 4,5-dicaffeoylquninic (130 mg) were isolated from PL using a Diaion Hp-20 resin column and Sephadex LH-20 column (Sigma Aldrich). These constituents were purified using a high-performance liquid chromatography-mass spectrometry system (Waters, Milford, MD, USA) under the following conditions: column, ACQUITY UPLC^®^ BEH C18 (1.7 μm, 2.1 mm × 100 mm, Waters); flow rate, 0.25 mL/min. The mobile phase consisted of (A) 0.1% formic acid and (B) acetonitrile. The following gradient elution was used: 0 min, 5% B; 5 min, 5% B; 25 min, 60% B; 30 min, 60% B. The constituents were identified using 1H and 13C nuclear magnetic resonance spectroscopy.

### 4.3. Preparation of Aβ_25–35_ Plaque

Aβ_25–35_ plaque was generated as described in a previous method [37]. Briefly, Aβ_25–35_ monomer was reconstituted in distilled water to a final concentration of 500 μmol/L. Aliquots were incubated at 37 °C for 3 days to make an amyloid plaque.

### 4.4. Free Radical Scavenging Assay

#### 4.4.1. ABTS Radical Cation Assay

ABTS solution (7.40 mM) was added to 2.60 mM potassium phosphate in the dark at room temperature 24 h before starting the experiment. Various concentrations of PL (2, 10, 50, and 250 μg/mL) were mixed with 7.40 mM ABTS solution and 2.60 mM potassium phosphate. After incubation at room temperature for 5 min, the absorbance of the mixture was measured at 732 nm using a spectrophotometer. Additionally, the antioxidant activity of PL was expressed as the half-maximal IC_50_ value. Values were estimated by nonlinear regression using Microsoft Excel (2016, Microsoft, Redmond, WA, USA): ABTS cation scavenging activity (%) = (control − sample)/control × 100.

#### 4.4.2. DPPH Radical Scavenging Assay

Radical scavenging activity was measured by a modified method [38]. Various concentrations of PL (2, 10, 50, and 250 μg/mL) were mixed with 0.20 mM DPPH ethanolic solution (1:1). The absorbance of the mixture was determined at 517 nm using a spectrophotometer. Antioxidant activity of PL was expressed as an IC_50_ value. Values were estimated by nonlinear regression using Microsoft Excel: DPPH radical scavenging activity (%) = (control − sample + blank)/control × 100.

### 4.5. Cell Culture and Treatment

Mouse hippocampal HT22 cells were obtained from Insug Kang at Kyung Hee University (Seoul, Korea) and cultured in DMEM supplemented with 10% heat-inactivated FBS and 1% PS in a water-saturated atmosphere of 5% CO_2_ incubated at 37 °C. The cells were subcultured 3 times a week. All experiments were carried out 18 h after cells had been seeded in 96-well plates and 60 mm dishes at densities of 2 × 10^3^ cells/well and 8 × 10^4^ cells/dish. After cell seeding, various concentrations of PL (1, 10, and 100 μg/mL) were applied to the cells for 24 h with or without 10 μmol/L Aβ_25–35_ plaque for the last 1 h of treatment.

### 4.6. Measurement of Cell Viability

Cell viability was determined using MTT assay [39]. Treated cells were incubated with 1 mg/mL MTT for 4 h. The supernatants were discarded, and the cells were dissolved in DMSO. Absorption was measured at 570 nm using a microplate reader (Molecular Devices, San Jose, CA, USA) and expressed as a percentage of the value of the control culture.

### 4.7. Measurement of Intracellular ROS

Intracellular ROS generation levels were measured using DCFH-DA reagent for fluorometric reaction. DCFH-DA is converted to form the fluorescent product 2′,7′-dichlorofluorescin (DCF) when ROS reacts with DCFH, a nonfluorescent compound. Treated cells were incubated with 20 μM DCFH-DA for 30 min. The fluorescence intensity was measured at 485 nm excitation and 535 nm emission using a fluorescence microplate reader (SpectraMax Gemini EM, Molecular Devices, Sunnyvale, CA, USA).

### 4.8. Western Blot Analysis

Western blot was carried out according to a previously described method [40]. Cell lysates were separated using 12% SDS-polyacrylamide gel electrophoresis and transferred to Immobilon-P Transfer Membrane (Millipore, Bedford, MA, USA). Membranes were incubated with 5% skim milk in TBST to block nonspecific binding protein and with the primary antibodies (1:2000 dilution of β-actin and HO-1; 1:1000 dilution of NQO1 and pCREB) overnight at 4 °C. Horseradish peroxidase-conjugated secondary antibodies were then incubated for 1 h. Immunoreactive bands were detected using enhanced chemiluminescence detection solution (Bio-Rad Laboratories, Hercules, CA, USA), and ChemiDoc^TM^ XRS+ (Bio-Rad Laboratories) was used for visualization. The intensities of the bands were normalized to the β-actin band using Image J (National Institutes of Health, Bethesda, MD, USA).

### 4.9. qRT-PCR Analysis

mRNA transcription of cytokines was analyzed by qRT-PCR. Total RNA was extracted from HT22 cells using the RNeasy Plus Mini Kit (Qiagen, Hilden, Germany), according to the manufacturer’s instructions. RNA samples (5 μg) were subjected to cDNA synthesis using an RNA to cDNA EcoDry Premix kit (Takara, Shiga, Japan). cDNA was subjected to qRT-PCR using SYBR Green Mix (Toyobo, Osaka, Japan) and the CFX Connect real-time PCR system (Bio-Rad Laboratories). Primers, synthesized at Cosmo Genetech (Seoul, Korea), were as follows: HO-1: forward, 5′-CAACAAGCAGAACCCAGTCT-3′, reverse, 5′-CTCGTGGAGACGCTTTACAT; NQO1: forward, 5′-AGATCCTGGAAGGATGGAAG, reverse, 5′-CACAGAGAGGCCAAACTTGT; GAPDH: forward, 5′-TGAATACGGCTACAGCAACA-3′, reverse, 5′-AGGCCCCTCCTGTTATTATG.

### 4.10. Animals

Male ICR mice (6 weeks, 27–30 g) were purchased from Daehan Biolink (Eumseong, Korea). Animals were accommodated at constant temperature (23 ± 1 °C) and humidity (60 ± 10%) and a 12 h light/dark cycle. They were housed and had free access to water and food. Animal treatment and maintenance were carried out in accordance with the Principles of Laboratory Animal Care (National Institutes of Health publication No. 85-23, revised 1985) and the Animal Care and Use Guidelines of Kyung Hee University, Seoul, Korea (approval number: KHUASP(SE)-15-083, 13 October 2015). We administered PL to mice orally for 17 days after intracerebroventricular (ICV) injection of Aβ_25–35_ plaque. Behavior tests were performed from day 13 to day 16 of PL treatment (Supplementary Figure S1). The operators responsible for the behavioral experimental procedure and data analysis were blinded and unaware of group allocation throughout the experiments.

### 4.11. Surgery Procedure

Mice were anesthetized and placed in a stereotaxic apparatus (myNeuroLab, St. Louis, MO, USA). Aβ_25–35_ plaque (15 nmol/L) was delivered by ICV injection, with coordinates −0.7 mm posterior and +1.2 mm lateral from the bregma and −2.0 mm ventral from the skull surface, according to the stereotaxic atlas of mouse brain [41]. The sham-operated mice were injected with the same volume of only saline. The accuracy of stereotaxic injection to the targeted region was monitored in all animals by examination of the needle tract within brain sections.

### 4.12. Behavior Test

#### 4.12.1. Novel Object Recognition Test

According to the method described previously, NORT was performed on day 13 of PL treatment. The test apparatus consisted of a dark open field box (45 cm × 45 cm × 45 cm). Prior to the test, mice explored the test apparatus without objects for 5 min daily for 5 days. After the habituation period, each mouse was placed into the test box with 2 identical objects and observed for 3 min. The time spent by the animal examining each object (defined as the training session) was measured. Twenty-four hours after the training session, mice were allowed to explore the objects in the test box for 3 min, using one of the training objects as well as a novel object. The time that the animals spent exploring the novel and familiar objects was measured (defined as the test session). The mice were considered to be interested when they were facing, sniffing, or touching the object. A memory index was expressed as percentage of novel object recognition time (time percentage = *t*-novel object/(*t*-novel object + *t*-familiar object) × 100).

#### 4.12.2. Passive Avoidance Test

On day 15 of PL treatment, learning and memory functions were measured using a 2-compartment step-through passive avoidance apparatus according to a previously described method [42]. The box was divided into bright (21 cm × 21 cm × 21 cm) and dark (21 cm × 21 cm × 21 cm) compartments by a guillotine door. The bright room contained a 50 W electric lamp, while the floor of the dark room was composed of 2 mm stainless steel rods spaced 1 cm apart. Treatment was carried out with PL or saline 1 h before the acquisition trial. Mice were then placed in the bright room for the acquisition trial. After 10 s, the door separating the chambers was opened. As soon as the mouse entered the dark room, the guillotine door was closed and an electrical foot shock (0.5 mA) was delivered throughout the grid floor for 3 s. Twenty-four hours after the acquisition trial, the mice were placed in the bright room for a retention trial. The latency time was defined as the time taken for a mouse to enter the dark room after the door opened. The latency time was recorded for up to 300 s.

### 4.13. Brain Tissue Preparation

At 24 h after the behavioral test, mice were anesthetized and perfused transcardially with 0.05 M PBS, and then fixed with cold 4% PFA in 0.1 M PB. Brains were removed and post-fixed in 0.1 M PB containing 4% PFA overnight at 4 °C. These were then immersed in a solution containing 30% sucrose in PBS for cryoprotection. Serial 30 μm thick coronal sections were cut on a freezing microtome (Leica, Nussloch, Germany) and stored in cryoprotectant (25% ethylene glycol, 25% glycerol, and 0.02 M PB) at 4 °C until use.

### 4.14. Immunohistochemistry

Brain sections were rinsed in PBS and treated with 1% hydrogen peroxide for 15 min. They were then incubated with mouse anti-NeuN antibody (1:1000 dilution), mouse anti-SYN antibody (1:250 dilution), rabbit anti-PSD-95 antibody (1:500 dilution), or mouse anti-pCREB (1:100 dilution) overnight at 4 °C in the presence of 0.3% triton X-100, 1% NGS, and 1% bovine serum albumin. Next, sections (NeuN, SYN, and PSD-95) were rinsed in PBS and incubated with biotinylated anti-mouse and anti-rabbit IgG (1:250 dilution) for 90 min and with ABC (1:100 dilution) for 60 min at room temperature. Peroxidase activity was visualized using DAB in 0.05 M tris-buffered saline (pH 7.6). After several rinses with PBS, sections were mounted on gelatin-coated slides, dehydrated, and cover-slipped using HistoMount medium. The optical densities of NeuN, SYN, and PSD-95-immunoreactivities in the CA3 or DG of the hippocampus were analyzed with Image J software. Brain sections (pCREB) were incubated with anti-mouse Alexa Fluor 594 (1:1000 dilution) for 60 min and mounted with anti-fade fluorescent medium. To measure the optical densities of NeuN, SYN, PSD-95, and pCREB, the entire region of interest was manually outlined and averaged optical densities were acquired in images with converted 8-bit indexed color. The images were photographed at 200× or 400× magnification using an optical light microscope (Olympus Microscope System BX51; Olympus, Tokyo, Japan) equipped with a 20× objective lens.

### 4.15. Statistical Analysis

All data were expressed as mean ± standard error of the mean (SEM) using Graph Pad Prism 5.0 (Graph Pad software, San Diego, CA, USA). The results were analyzed statistically by unpaired *t*-test or one-way analysis of variance followed by Tukey’s post hoc test. A *p*-value less than 0.05 was considered statistically significant.

## 5. Conclusions

We first demonstrated the neuroprotective effects of PL in Aβ_25–35_ plaque-induced AD models. PL protects hippocampal cells against Aβ_25–35_ plaque toxicity by regulating intracellular ROS levels and CREB-induced activation of antioxidant enzymes in in vitro. Administration of PL ameliorated memory impairment as well as neuronal cell damage and synaptic disruption in Aβ_25–35_ plaque-infused AD mice. Together, our results suggest that PL could be a useful candidate for slowing progression or preventing onset of AD.

## Supplementary Materials

Supplementary materials can be found at http://www.mdpi.com/1422-0067/19/6/1644/s1.
