# Supplementary material for: Butterbur Leaves Attenuate Memory Impairment and Neuronal Cell Damage in Amyloid Beta-Induced Alzheimer’s Disease Models
Source: Int J Mol Sci. 2018 Jun 1;19(6):1644. doi: 10.3390/ijms19061644 (PMC6032106; doi:10.3390/ijms19061644)
Supplement: Supplementary file 1 [file ijms-19-01644-s001.pdf]

Supplementary Materials: Butterbur Leaves Attenuate Memory Impairment and Neuronal Cell Damage in Amyloid Beta-Induced Alzheimer's Disease Models

Namkwon Kim, Jin Gyu Choi, Sangsu Park, Jong Kil Lee and Myung Sook Oh

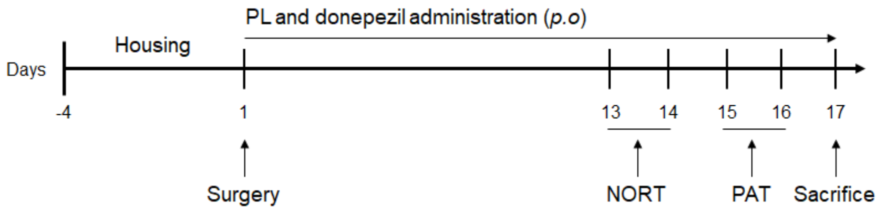

Figure S1. Experimental protocol. PL; *Petasites japonicus* leaves, NORT; novel object recognition test, PAT; passive avoidance test.
